# Get real: Orbitofrontal cortex mediates the ability to sense reality in early adolescents

**DOI:** 10.1002/brb3.1552

**Published:** 2020-02-19

**Authors:** Maria Chiara Liverani, Lorena G. A. Freitas, Vanessa Siffredi, Greta Mikneviciute, Roberto Martuzzi, Djalel‐Eddine Meskaldij, Cristina Borradori Tolsa, Russia Ha‐Vinh Leuchter, Armin Schnider, Dimitri Van De Ville, Petra Susan Hüppi

**Affiliations:** ^1^ Department of Paediatrics, Gynecology and Obstetrics Division of Development and Growth Geneva University Hospitals Geneva Switzerland; ^2^ Institute of Bioengineering École Polytechnique Fédérale de Lausanne Lausanne Switzerland; ^3^ Foundation Campus Biotech Geneva Geneva Switzerland; ^4^ Institute of Mathematics École Polytechnique Fédérale de Lausanne Lausanne Switzerland; ^5^ Department of Clinical Neurosciences Division of Neurorehabilitation Geneva University Hospitals Geneva Switzerland

**Keywords:** early adolescents, fMRI, memory, orbitofrontal cortex, orbitofrontal reality filtering

## Abstract

**Introduction:**

Orbitofrontal reality filtering (ORFi) is a memory mechanism that distinguishes whether a thought is relevant to present reality or not. In adults, it is mediated by the orbitofrontal cortex (OFC). This region is still not fully developed in preteenagers, but ORFi is already active from age 7. Here, we probe the neural correlates of ORFi in early adolescents, hypothesizing that OFC mediates the sense of reality in this population.

**Methods:**

Functional magnetic resonance images (fMRI) were acquired in 22 early adolescents during a task composed of two runs: run 1 measuring recognition capacity; run 2 measuring ORFi; each containing two types of images (conditions): distractors (D: images seen for the first time in the current run) and targets (T: images seen for the second time in the current run). Group region of interest (ROI) analysis was performed in a flexible factorial design with two factors (run and condition) using SPM12.

**Results:**

We found significant main effects for the experimental run and condition. The bilateral OFC activation was higher during ORFi than during the first run. Additionally, the OFC was more active while processing distractors than targets.

**Conclusion:**

These results confirm, for the first time, the role of OFC in reality filtering in early adolescents.

## Graphical abstract

Orbitofrontal reality filtering (ORFi) is a memory mechanism that distinguishes whether a thought is relevant to present reality or not, and in adults it is mediated by the orbitofrontal cortex (OFC). In children, the OFC is one of the last regions to develop, but ORFi is already functional from age 7. Using functional magnetic resonance images, we probe for the first time the neural correlates of ORFi in early adolescents, hypothesizing that OFC mediates the sense of reality in this population.

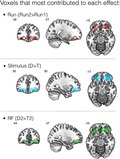

## 1 INTRODUCTION

Orbitofrontal reality filtering (ORFi) is a memory control mechanism that allows to filter upcoming memories and thoughts according to their relation with ongoing reality (Schnider, 2003, 2018). The first description of ORFi was based on the observation of patients with orbitofrontal lesions, suffering from behaviorally spontaneous confabulations and disorientation. These patients typically act to currently inappropriate memories to guide their present actions or to shape their future plans, failing to verify the connexion of these memories with the “now.” In addition, they are disoriented in time and space (Schnider, 2018). For example, a retired psychiatrist hospitalized after rupture of an aneurysm of the anterior communicating artery, repeatedly tried to leave the hospital in the conviction that she had to meet her own patients (Schnider, Bonvallat, Emond, & Leemann, 2005).

Schnider and colleagues (Schnider, von Daniken, & Gutbrod, 1996) developed an experimental paradigm to test ORFi and to reliably discriminate reality‐confusing patients from healthy participants. It consists of two runs of a continuous recognition task in which the same images are shown twice. Participants are asked to indicate picture recurrences only within the ongoing run. The first run assesses the ability to encode and recognize items, and familiarity alone is sufficient to correctly perform the task. In the second run all images are familiar, and thus familiarity alone is not enough to perform the task. In this second run, ORFi is needed, representing the ability to sense whether the memory of an item relates to the present (the currently ongoing run), or not (Schnider & Ptak, 1999).

Behaviorally, confabulating patients markedly and specifically increased their false positives in the second run (Nahum, Bouzerda‐Wahlen, Guggisberg, Ptak, & Schnider, 2012; Schnider & Ptak, 1999). Lesion analysis on these patients revealed that the ORFi mechanism depends on the orbitofrontal cortex (OFC) or structures directly connected with it (Schnider, 2018; Schnider et al., 1996). Functional neuroimaging studies using positron emission tomography further corroborated the dependence of ORFi on the intact OFC (Schnider, Treyer, & Buck, 2000; Treyer, Buck, & Schnider, 2003, 2006). Electrophysiological studies revealed that ORFi is expressed by a frontal positivity at about 200–300 ms, before the content of a thought is recognized (Schnider, Valenza, Morand, & Michel, 2002).

Children are more vulnerable to memory distortions and more prone to errors than adults (Ceci & Bruck, 1993). Using a child‐adapted version of the continuous recognition task, we recently found that ORFi is present in 7‐year‐old children, improves from 7 to 11 years in parallel with memory capacity, but does not attain adult efficacy at that age (Liverani et al., 2017).

The neural correlates of this mechanism in children and adolescents have never been investigated. While the implication of the OFC in ORFi has clearly been shown in adults (Treyer, Buck, & Schnider, 2003, 2006), no such evidence exists in a younger population.

The aim of this study was to examine, with advanced functional neuroimaging techniques, to which extent the ability of early adolescents to sense whether a memory or a thought refers to the present reality or not depends on the OFC, similar to what has been found in adults.

## 2 METHODS

### 2.1 Participants

Twenty‐three healthy early adolescents from 10 to 13 years of age (10 females, mean age 12 ± 1.01 years) were recruited through advertisements. One participant was excluded due to strong signal distortions on fMRI images caused by the subject's dental braces. Twenty‐two participants were finally included in the analysis.

Cognitive assessment at the time of the scan was performed using the French version of the Wechsler Intelligence Scale for Children—Fifth Edition (WISC—V, Wechsler, 2014). For one participant, IQ score was evaluated using the Kaufman Assessment Battery for Children, Second Edition (KABC‐II, Kaufman & Kaufman, 2004). All participants scored within the normal range of intellectual functioning (mean = 117.04 ± 11–35). Parents were asked to fill a questionnaire assessing the presence of serious physical illness or neurological problems. None of the participants had major disabilities, psychiatric, or neurological diseases.

The Ethics Committee of the Canton of Geneva approved the study, which was carried out in accordance with the Declaration of Helsinki. Caregivers and participants provided informed written consent. Participants received a gift voucher of 100 Swiss francs for their participation in the study.

### 2.2 fMRI paradigm

**Figure 1:**
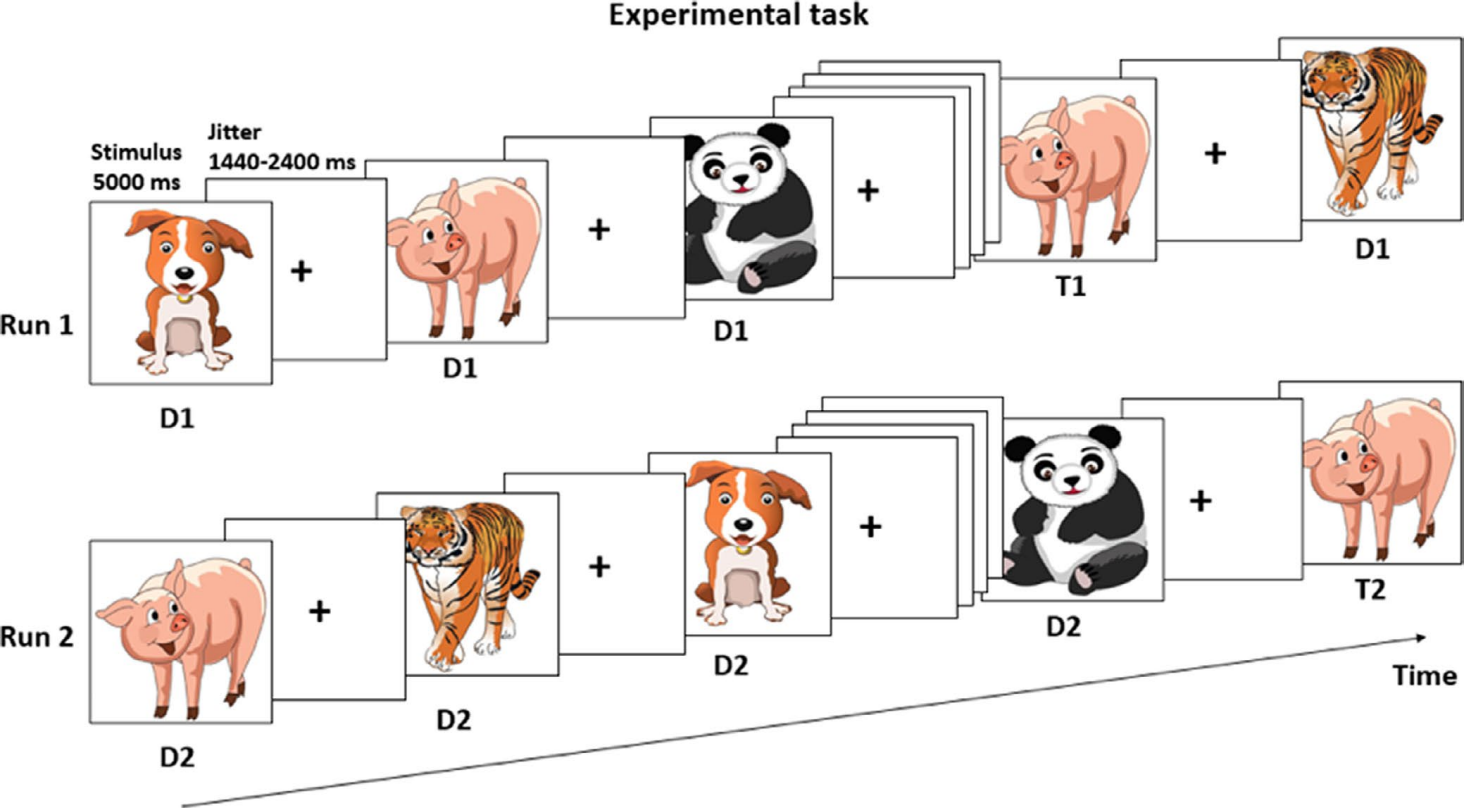
Task design. The task was composed of 2 runs, separated by a break of 3 min. Distractors (D1 and D2) are images presented for the first time within a run; targets (T1 and T2), are images repeated within the same run

Participants performed a child‐adapted version of the continuous recognition task assessing recognition memory and orbitofrontal reality filtering (Figure 1; Liverani et al., 2017; Schnider, 2003, 2013; Schnider et al., 1996), associated with an event‐related fMRI paradigm.

The task was composed of two runs in which the same set of images was presented and repeated twice, with a break of around 3 min between the two runs. In the first part, assessing recognition memory (item recognition, IR) participants were asked to indicate picture recurrence ("Have you already seen this picture in this task?”) by pressing the left button of an MRI‐compatible mouse if the image was seen for the first time (distractors run 1, D1), and the right button if it was seen for the second time (targets run 1, T1). This run can be solved on the basis of familiarity alone. In the second run, the same set of pictures was presented in a different order and repeated twice. Participants were instructed to indicate whether each item was presented for the first or the second time in this ongoing run ("Is this the first or the second time that you see that image in this ongoing run?"), pressing the left button of the mouse for images seen for the first time (distractors run 2, D2), and the right button for images presented for the second time (targets run 2, T2). In this run, all images have already been seen. Therefore, familiarity alone is not enough to correctly perform the task, and the ORFi mechanism is needed to process distractors (D2).

Pictures were a set of 30 cartoon drawings of animals and were presented for 5 s on the screen. In each run, 30 images were presented for the first time (distractors, D) and then repeated once (targets, T) after 6–9 intervening pictures, as already done in a previous study with children (Liverani et al., 2017). After each image, a fixation cross was presented during between 1,440 and 2,400 milliseconds. Each run lasted approximately 7.5 min. Stimuli were displayed on a white screen at the head of the scanner via a 45° angled mirror fixed to the MRI head coil. Responses were given by pressing two buttons with the right index and middle finger, on an MRI‐compatible mouse. Task programming, stimuli display, and responses logging were done using E‐Prime 2 (Psychology Software Tools, Pittsburg, USA). All participants successfully completed a short training with a different set of images in the mock MRI scanner before the MRI.

### 2.3 Behavioral data analysis

Reaction times and accuracy were recorded for each condition (D1, T1, D2, T2). A 2 × 2 repeated measures analysis of variance (ANOVA) was performed on accuracy and reaction time with the within‐subject factors run (1, 2) and stimulus (distractor D, target T).

### 2.4 Image acquisition

MRI data were acquired on a Siemens 3T Magnetom Prisma scanner at Campus Biotech, Geneva, Switzerland. Structural T1‐weighted MP‐RAGE (magnetization‐prepared rapid gradient‐echo) sequences were acquired using the following parameters: voxel size = 0.9 × 0.9 × 0.9 mm; repetition time (TR) = 2,300 ms; echo time (TE) = 2.32 ms; inversion time (TI) = 900 ms; flip angle (FA) = 8°; and field of view (Fov) = 240 mm. Functional images were T2*‐weighted with a multislice gradient‐echo‐planar imaging (EPI) sequence of 64 slices; voxel size = 2 × 2 × 2 mm; TR = 720 ms; TE = 33 ms; and Fov = 208 mm. Finally, a fieldmap was acquired each time a participant entered the scanner, with TR = 627 ms; TE_1_ = 5.19 ms; TE_2_ = 7.65 ms; and FA = 60°.

### 2.5 MRI data preprocessing

Our data were preprocessed using SPM12 (Wellcome Department of Imaging Neuroscience, UCL, UK) in Matlab R2016a (The MathWorks, Inc., Natick, Massachusetts, United States). One particular challenge in studying frontal brain areas using fMRI is the considerable vulnerability of these regions to signal distortions caused by field inhomogeneities around the air‐filled sinuses (Gorno‐Tempini et al., 2002). To correct for the resulting geometrical distortions, a field map was calculated from an additional stock double‐echo field map sequence included in our MRI protocol (Hutton et al., 2002). The fMRI images from each participant were then spatially realigned and unwarped, respectively, to correct for motion artifacts and potential geometric distortions. Thanks to the distortion correction of vulnerable brain regions on the single‐subject level, this additional unwarping step not only improves the coregistration between structural and functional images, but it also reduces the distortion variability across subjects during spatial normalization to a common space (Hutton et al., 2002). This solution has been successfully used in several recent studies in adults including task (Daw, Gershman, Seymour, Dayan, & Dolan, 2011) and resting‐state (Togo et al., 2017) experimental paradigms, as well as in presurgical planning (Cardoso et al., 2018) and in children (Wozniak et al., 2011).

In general, total head motion was very low on our participants as measured by framewise displacement (FD; Power et al., 2014): for the first fMRI run the mean FD per frame was 0.16 mm with a standard deviation (*SD*) of ±0.04 mm; for the second run the mean FD was 0.15 mm ± 0.05 mm. Therefore, no participant was excluded due to high motion. Functional images were then coregistered to structural images in subject space and smoothed with a Gaussian filter of full width at half maximum (FWHM) = 6 mm. To be able to perform a group level comparison, data were warped into MNI (Montreal Neurologic Institute) space via a study‐specific DARTEL (Diffeomorphic Anatomical Registration using Exponentiated Lie algebra) template. Normalization methods such as these have been demonstrated to be robust to age differences in participants of 7 years and above (Ashburner & Friston, 1998; Burgund et al., 2002). Additionally, the inclusion of the DARTEL template as an intermediate step is among the top ranked currently available deformation algorithms (Klein et al., 2009).

### 2.6 Region of Interest (ROI) analysis

Statistical analyses were performed using SPM12 scripts implemented in Matlab R2016a in a two‐step process, so that both intra‐ and inter‐subject variances were taken into account (Friston, Frith, Frackowiak, & Turner, 1995). First‐level (subject level) analyses were assessed on a voxelwise basis using a General Linear Model (GLM). Within each condition, trials responded correctly and incorrectly were pooled together to generate the corresponding regressors. This was motivated by two main reasons: (a) This would ensure a similar number of trials per condition, and (b) our participants had consistently high rates of correct responses, which characterizes a ceiling effect as discussed later. The condition regressors were produced by convolving SPM12's canonical hemodynamic response function (HRF) with the onsets of each trial in an event‐related design and included as regressors of interest in the individual design matrix. To further account for potential individual movement effects, we included in our model covariates of no interest calculated in the following fashion: First, we computed the 24‐parameter Volterra expansion (VE) of the 6 motion parameters stored during the realignment step of the preprocessing pipeline. Secondly, we extracted the top 6 components (or those that explained 95% of the variance in the VE) via singular value decomposition (SVD). Then, we included these components as nuisance regressors in the subject‐level design matrix. This approach has been successfully used on our previous analyses of child data (see Adam‐Darque et al., 2018 for an example). Finally, we employed the scan‐nulling strategy (Lemieux, Salek‐Haddadi, Lund, Laufs, & Carmichael, 2007) to ignore information contained in fMRI images in which FD > 0.5 mm, by adding extra regressors of no interest for each of these time points.

**Figure 2:**
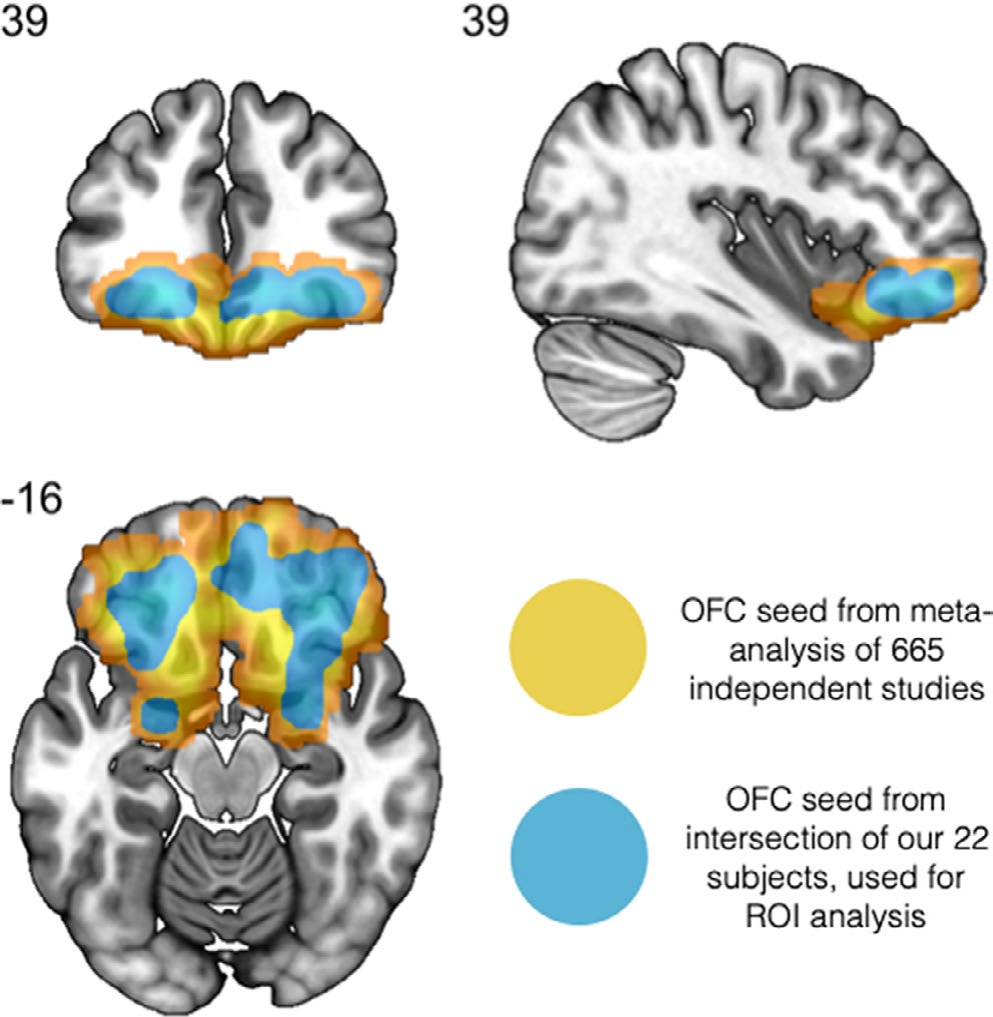
The shaded areas show the Orbitofrontal Cortex ROI. The region highlighted in yellow indicates the initial mask calculated from 665 independent studies using NeuroSynth. The area highlighted in blue corresponds to the intersection of gray matter voxels available for all participants within the initial mask. The latter was the final ROI used for this study. Brain images follow the neurological convention (left side shown on the left; right side shown on the right)

The first‐level results from all participants were then used in a second‐level (group level) analysis in a factorial design with two factors (run and condition) with two levels each (2 runs and 2 types of stimuli, namely distractor and target). This design provides the flexibility to analyze main effects as well as a possible interaction effects between the factors. Given the a priori hypothesis of the involvement of the OFC in the reality filtering task based on neuropsychological data, lesion studies, and PET imaging studies (Schnider et al., 1996; Schnider & Ptak, 1999; Treyer, Buck, & Schnider, 2003), we performed an ROI analysis based on this brain region. Our ROI mask was defined as follows: First, we downloaded a z‐scored mask from NeuroSynth (Wager, 2011), which was calculated as a meta‐analysis of 665 independent studies for the term “orbitofrontal cortex.” This initial mask (nMask) was thresholded at *z*‐value > 3, which is equivalent to a *p*‐value < .001, and the largest continuous cluster was maintained. The nMask covered the entire bilateral OFC and can be seen highlighted in yellow in Figure 2. Last, to ensure an equal contribution of all subjects to the analysis, we created a final mask (iMask) calculated as the intersection of all voxels within nMask that were present in the gray matter of every subject in our dataset. This can be seen as the blue highlight in Figure 2. The contrast values for voxels within the ROI iMask from each subject were then averaged, and the resulting value entered in a 2‐way analysis of variance (ANOVA). This strategy has two main advantages: It increases the signal to noise ratio, which improves the power of detecting true signals, and avoids the problem of multiple testing inherent in massive univariate approaches (Benjamini & Heller, 2007; Meskaldji et al., 2015). Although the ANOVA allows us to identify main, as well as interaction effects, it does not describe the effect's direction—for example, it may tell us that the means between conditions are different, but not which one is greater. Thus, we performed additional *t* tests within factors to clarify the direction of the effects found with the ANOVA and report the corresponding *p*‐values, Bonferroni corrected for the number of effects that we find. Furthermore, in order to provide an estimate of each voxel's contribution to the effects detected by these tests, we calculated the voxelwise *t*‐values within the ROI.

## 3 RESULTS

### 3.1 Behavioral results

Behavioral descriptive results on accuracy and reaction times are summarized in Table 1. Overall, a ceiling effect was found for the task accuracy, since participants had a very high rate of correct responses. The 2 × 2 repeated measures ANOVA on reaction times revealed a significant main effect of the factor run (*F*_(1,21)_ = 12.14, *p* < .005, $\eta_{p}^{2}$ = 0.366), with faster responses for the first compared to the second run. No significant difference was found between Distractors and Targets reaction time (*F*_(1,21)_ = 0.001, *p* = .977, $\eta_{p}^{2}$ = 0.000). The interaction between the factor run and the factor Condition was not significant.

**Table 1:** Descriptive statistics of behavioral results on the Reality Filtering task

Stimulus type	Correct responses, % (*SD*)	Reaction times, ms (*SD*)
Distractor, run 1	96.06 (4.78)	1,454 (406)
Target, run 1	90.30 (16.93)	1,454 (369)
Distractor, run 2	93.63 (6.24)	1,579 (339)
Target, run 2	89.39 (13.97)	1,577 (413)

Note

Distractor, run 1 and Distractors, run 2 are images seen for the first time in the first and in the second run, respectively. Target, run 1 and Target, run 2 are images seen for the second time in the first and in the second run, respectively.

**Figure 3:**
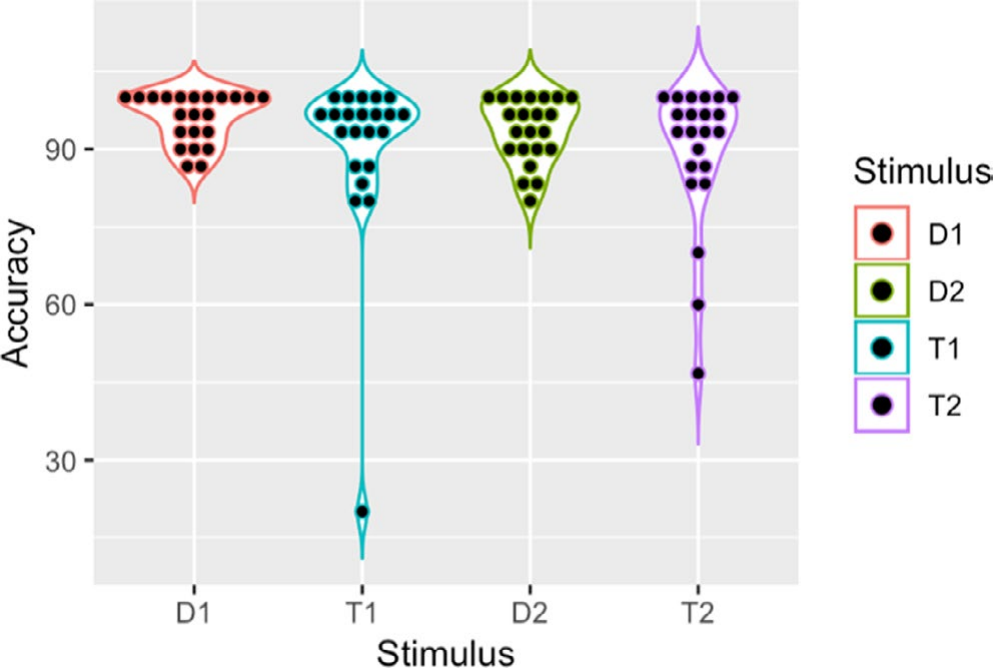
Violin plot showing accuracy distribution per stimulus in the population

**Figure 4:**
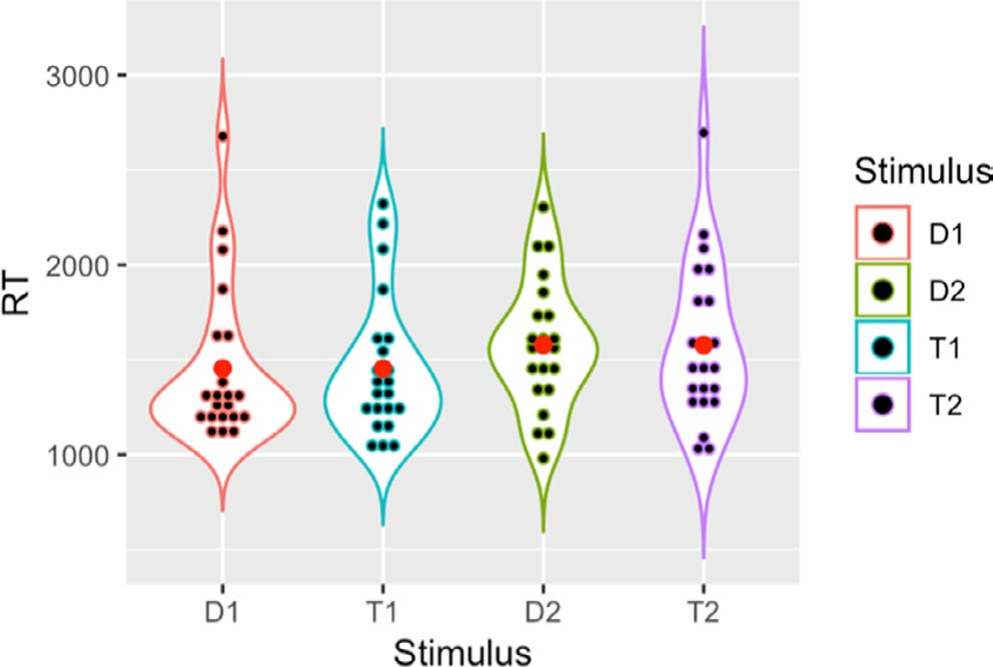
Violin plot showing reaction time distribution per stimulus in the population

Accuracy analysis revealed no difference between the two runs (*F*_(1,21)_ = 1.36, *p* = .257, $\eta_{p}^{2}$ = 0.061), as well as no difference between Distractors and Targets (*F*_(1,21)_ = 3.14, *p* = .91, $\eta_{p}^{2}$ = 0.13). The interaction between the factor Run and the factor Condition was not significant. Violin plots in Figures 3 and 4 show the distribution of correct responses and reaction times for each condition, respectively.

### 3.2 ROI task‐related activity

To investigate whether there were main effects of run or condition, or an interaction between the two in the OFC, we first ran a 2‐way ANOVA test (see Table 2). We found a significant main effect for the experimental “run” (*F*_(1,21)_ = 556.65, *p* = .027). Additionally, we found a significant main effect of the factor “condition” (*F*_(1,21)_ = 1,014.64, *p* = .02). The interaction effect between run and condition was nonsignificant.

**Table 2:** 2‐way ANOVA with factors "run" and "condition" for brain activations in the OFC

Factors	Mean squared	*F*	*p*‐value
run	1.3219	556.65	.027
condition	2.4095	1,014.64	.02
run * condition	0.0024	0	.9455

Note

run = run 1 and run 2; condition = Distractors and Targets.

**Figure 5:**
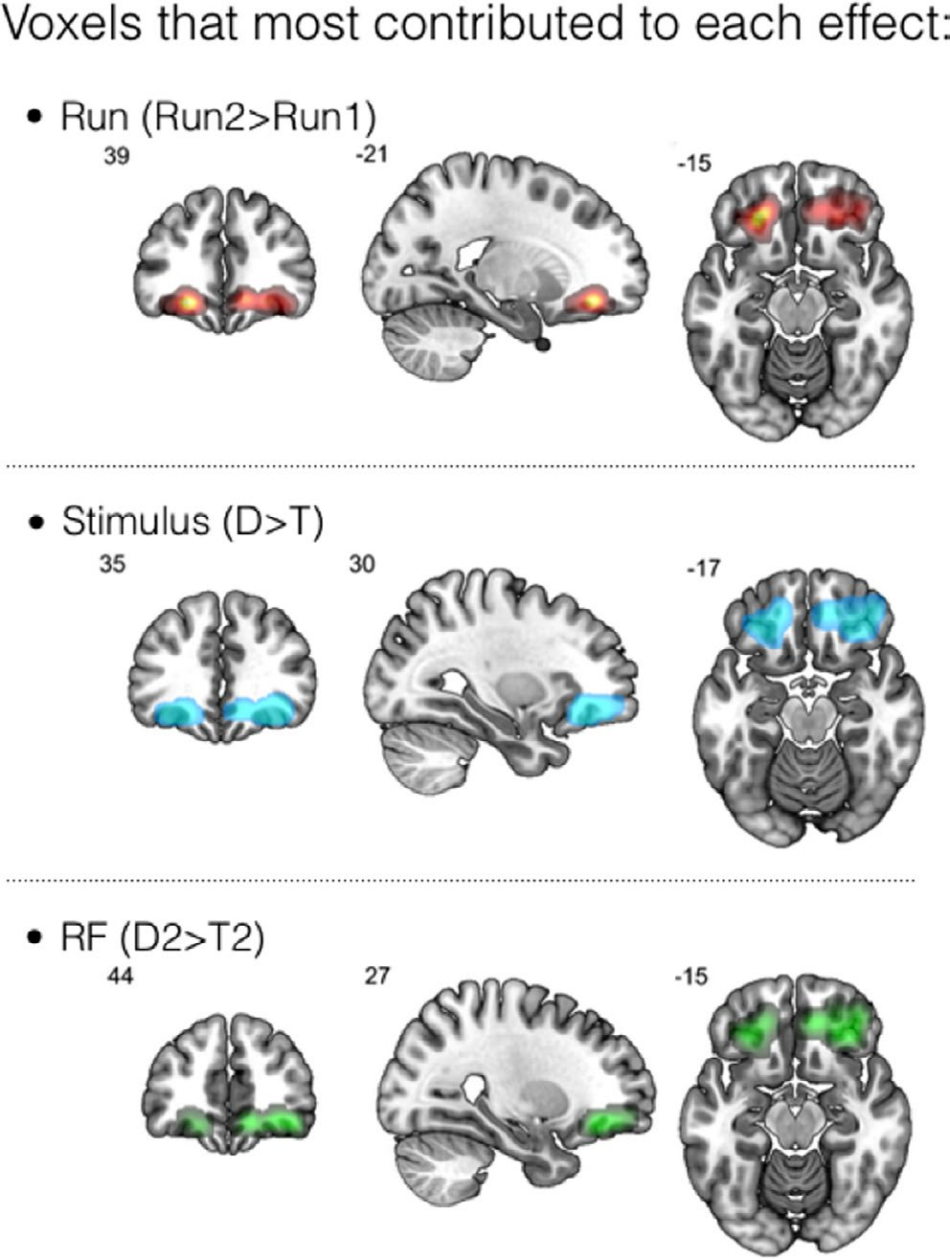
Contribution of OFC voxels to each effect. Brighter colors indicate a stronger contribution

We next sought to clarify the direction of the effects found from the ANOVA test. To this end, we first carried out a *t* test comparing run 2 and run 1 (see Table 3). As we hypothesized that the mean activation of the OFC during run 2 would be higher than during run 1, we first performed a one‐tailed test. Indeed, we found that the overall bilateral OFC activation was higher during the run 2, which specifically assess the reality filtering mechanism, than during run 1 (*T*_(21)_ = 2.12, *p*_(bonf)_ = .04). Secondly, we performed a one‐tailed *t* test to compare the condition levels, with the hypothesis that the OFC would present a higher activity while processing distractors (D) than targets (T) all run 1 and run 2 together. This effect was also highly significant (*T*_(21)_ = 3.70, *p*_(bonf)_ = .0006). The comparison between D2 and T2 (distractors and targets from the second run, respectively) showed that their means were also significantly different in the same direction (*T*_(21)_ = 2.41, *p* = .01). Figure 5 shows the voxelwise contribution to these results.

**Table 3:** Post hoc *t* tests on activation in the OFC

Comparison	*t*‐value	*p*‐value
run 2 > run 1	2.1172	.04
D > T	3.7002	.0006
D2 > T2	2.41	.01

Note

D = Distractors, T = Targets, D2 = Distractors of run 2, T2 = Targets of run 2.

## 4 DISCUSSION

With this study, we assessed for the first time in a young population and using fMRI, the neural correlates of ORFi, a memory control mechanism crucial to maintain thoughts and behavior in phase with reality.

Behaviorally, participants performed the test without difficulties and no differences in the accuracy were found, neither between the two types of stimuli (Distractors and Targets) nor between the two runs (1, 2). Moreover, the majority of participants performed well, making very few errors. This is similar to healthy adults, who had no difficulties to correctly perform the task even when runs were separated by only 1 min (Schnider & Ptak, 1999; Wahlen, Nahum, Gabriel, & Schnider, 2011). Our results corroborate the idea that at this age ORFi is already an intuitive and efficacious cognitive process, corresponding to the storage capacity at that age (Liverani et al., 2017). Regarding reaction times, responses were slower in the second run of the task compared to the first run, reflecting the main challenge of the task, which is consistent with previous studies (Bouzerda‐Wahlen, Nahum, Liverani, Guggisberg, & Schnider, 2015; Liverani, Manuel, Guggisberg, Nahum, & Schnider, 2016; Liverani et al., 2017). It appears that distinguishing between memories that are pertinent with the ongoing reality or not is more time consuming and takes more cognitive effort than simply recognizing previously seen images. Our previous study assessing orbitofrontal reality filtering in children highlighted a significant difference between distracters and targets both for accuracy and reaction times (Liverani et al., 2017). In the current, study participants were older, and they managed to distinguish almost perfectly between images seen in the current or in the previous run, performing at ceiling effect. Therefore, this could explain why no differences in accuracy and reaction time have been found between the two conditions.

Orbitofrontal cortex activation was significantly stronger during the second run, which tests ORFi. Thus, our neuroimaging data in early adolescents were in line with lesion and imaging studies in adults, indicating that in this younger population, like in adults, the ORFi mechanism is needed to accomplish the second run of the task, and it is associated with specific OFC activation. Moreover, compared to Targets, OFC activation significantly increases in response to Distractors, stimuli that specifically require ORFi. Thus, using fMRI to explore ORFi for the first time, we confirmed previous findings showing that the ability to select information pertaining to the ongoing reality and to suppress irrelevant memory traces is associated with the activation of the OFC.

Another added value of our study is that it extends these findings to a younger population: early adolescents aged between 10 and 13. Adolescence is a critical period in the development of the prefrontal cortex. There is a general consensus that the OFC—and the whole PFC—reaches complete maturity only at 20 years of age or more (Diamond, 2002; Galvan et al., 2006; Gogtay et al., 2004). Gray matter volume in the prefrontal cortex attains its maximal volume between 11 and 12 years old and then starts to decrease (Giedd et al., 1999), with a parallel improvement in cognitive functions such as source memory (Sowell, Delis, Stiles, & Jernigan, 2001). Given the late development of these prefrontal regions, one might speculate that the neural substrates of certain cognitive functions differ from early adolescence to adulthood. Nevertheless, our findings showing OFC activation while performing the reality filtering task in early adolescents of 10–13 years old indicate that this brain structure has matured enough to assume this function.

The filtering of current irrelevant memories—that is, ORFi—bears conceptual resemblance with inhibitory control, defined as the ability to deliberately inhibit dominant, automatic, or prepotent responses that are currently irrelevant (Harnishfeger, 1995; St Clair‐Thompson & Gathercole, 2006). According to Schnider (2018), ORFi does not effectively “inhibit” memories that are not pertinent with the ongoing reality, but it adapts their format, labelling, and differentiating them as “fantasy” or “reality.” This process allows healthy individuals to then act differently and adequately according to fantasies or daydreams (Nahum, Ptak, Leemann, & Schnider, 2009; Schnider, 2018). Behavioral and neuroimaging data support this dissociation between ORFi and inhibitory control. Firstly, the ability to reject memories that are irrelevant to the present moment is already effective at the age of 7 (Liverani et al., 2017) and does not correlate with behavioral inhibition measures, which is one of the last high‐order functions to develop, continuing to consistently improve during adolescence (Luna, Padmanabhan, & O'Hearn, 2010). Secondly, the present study confirms that the neural basis of ORFi resides in the OFC already in 10 years old early adolescents. This finding corroborates the anatomical dissociation between the two mechanisms, since inhibition of unwanted memories has been associated with the activation of other prefrontal regions, such as dorsolateral prefrontal cortex, inferior frontal gyrus, and medio‐temporal lobe (Anderson et al., 2004; Luna et al., 2010).

In addition to being separate from inhibition processes, ORFi also needs to be differentiated from another memory‐monitoring ability, called source monitoring. Source monitoring is defined as the ability to accurately verify under which circumstances a memory has been acquired, and if it was self‐generated or not (Mitchell & Johnson, 2009). Previous studies demonstrated a behavioral and electrophysiological dissociation between the two mechanisms (Bouzerda‐Wahlen et al., 2015). Behaviorally, the retrieval of the source of a memory is a more demanding process compared to ORFi, as indicated by slower reaction times and higher error rates. Electrophysiologically, ORFi is characterized by a frontal positivity at 200–300 ms, while source monitoring is associated with a prolonged positivity from 400 ms on (Bouzerda‐Wahlen et al., 2015). Unlike ORFi, the developmental trajectory of source monitoring is unclear: Young children may be more prone than adult to confuse memories from different sources (Lindsay, Johnson, & Kwon, 1991), but the debate is still open. Anatomically, different brain areas participate in source monitoring, including the precuneus (Lundstrom, Ingvar, & Petersson, 2005), the medial temporal lobe (Ross & Slotnick, 2008), and the prefrontal cortex (Mitchell & Johnson, 2009; Mitchell, Johnson, Raye, & Greene, 2004) but not the OFC, specifically. Even if more whole‐brain exploratory analyses would be needed, our results indicate a distinct activation pattern between ORFi and source monitoring. This corroborates the idea of the existence of two separate memory‐monitoring mechanisms that dissociate at the behavioral, anatomical, and electrophysiological level.

Given the crucial importance of ORFi for the correct adaptation of behavioral demands in everyday life, it is of major interest to better investigate what is the impact of a deficit in this mechanism in other clinical populations characterized by lesions or atypical development in the OFC region. One promising field of research concerns schizophrenia, a psychiatric condition associated with loss of gray matter in this region. Indeed, recent studies showed that an abnormal ORFi can be an early biomarker of schizophrenia spectrum disorder (Theze et al., 2019). Another population characterized by specific alteration in the OFC region is premature children (Gimenez et al., 2006). Up to now, no studies assessing the function of the OFC in the context of preterm birth have been done. Future research should address this point, using the paradigm assessing ORFi as a reliable task to explore OFC functions in premature children and adolescents.

## 5 CONCLUSION

This research investigated for the first time using fMRI technique the neural correlates of orbitofrontal reality filtering in early adolescents. Results showed that, as in adults, the orbitofrontal cortex is the region responsible to filter memories and thoughts according to their relevance to the now in this young population.

## CONFLICT OF INTEREST

We wish to confirm that there are no known conflicts of interest associated with this publication and there has been no significant financial support for this work that could have influenced its outcome.

## Data Availability

The data that support the findings of this study are available from the corresponding author upon reasonable request.
